# Supplementary material for: Isolation of Angola-like Marburg virus from Egyptian rousette bats from West Africa
Source: Nat Commun. 2020 Jan 24;11:510. doi: 10.1038/s41467-020-14327-8 (PMC6981187; doi:10.1038/s41467-020-14327-8)
Supplement: Supplementary file 1 — Supplementary Information [file 41467_2020_14327_MOESM1_ESM.pdf]

Isolation of Angola-like Marburg virus from Egyptian rousette bats from West Africa

Amman et al.

Supplementary Table 1. Summary table of MARV positive tissues and sequences
Summary of MARV positive tissues and MARV sequences obtained from bats in Sierra Leone.

					Sequence			
**Bat Sequence ID**	**Sw/Bl**	**Li/Spl**	**LN**	**Sal Gl**	**NP**	**VP35**	**Genome**	**Method**
343 Kasbat_SL_2018	--	--	35.71	--	yes	yes	no	Minion
345 Kasbat_SL_2018	--	34.88	--	--	yes	yes	no	Minion
417 Kasbat_SL_2018	--	--	35.28	--	yes	no	no	Sanger
940 Kasbat_SL_2018	--	--	35.11	--	yes	yes	no	Sanger
942 Kasbat_SL_2018	--	35.85	31.67	--	no	no	no	Sanger
960 Kasbat_SL_2018	--	30.07*‡	29.02*‡	30.59	yes	no	yes‡	MiSeq
965 Kasbat_SL_2018	--	31.05	--	--	yes	yes	no	Sanger
968 Kasbat_SL_2018	--	28.22*	--	--	yes	no	yes	MiSeq
1000 Kasbat_SL_2018	--	29.94*	--	--	yes	no	yes	MiSeq
SLAB3960Kakbat SL 2017	yes	34.0	--	--	yes	yes	yes	Illumina, Sanger
SLAB4104Koebat SL 2017	yes	37.0	--	--	yes	yes	yes	Sanger

A Ct value with an * indicates isolation positive. Positive qRT-PCR samples are shown with Ct values: Swab = oral swab; Li/Spl = pooled liver and spleen; LN = axillary lymph node; Sal Gl = salivary gland.
* Marburg virus isolation positive tissue
‡ Whole genome sequence generated from two tissues of one bat

Supplementary Table 2. Pairwise comparisons of select marburgvirus sequences
Genome pairwise comparisons of select marburgvirus sequences representing each of the major phylogenetic clades.

	**1**	**2**	**3**	**4**	**5**	**6**	**7**	**8**	**9**	**10**	**11**	**12**
**1**												
**2**	99.3											
**3**	79.1	79.0										
**4**	78.6	78.5	92.1									
**5**	78.9	78.8	92.6	95.8								
**6**	79.1	78.9	92.8	96.1	98.9							
**7**	79.2	79.1	93.1	96.5	98.2	98.5						
**8**	78.9	78.8	94	91.9	92.3	92.5	92.9					
**9**	79	78.9	93.8	91.6	92	92.2	92.6	97.8				
**10**	78.9	78.9	92.9	91.2	91.7	91.9	92.2	92.7	92.3			
**11**	78.9	78.8	93.2	91.5	92	92.3	92.5	92.9	92.6	96.4		
**12**	79.1	79.0	93.1	91.5	91.9	92.2	92.4	92.8	92.4	97.2	96.6	

1: Rav Ken 1980, 2: 982 bat Uga 2008, 3: Musoke Kenya 1980, 4: SLAB4104Koebat SL 2017, 5: 371 bat Uga 2007, 6: 01DRC 1999, 7: Oz Zim 1975, 8: Popp Uga/Ger 1967, 9: 422Kabale Uga 2012, 10: SLAB3960Kakbat SL 2017, 11: 1379c Ang 2005, 12: 1000Kasbat SL 2018
Lightface type: % nucleotide identity

Supplementary Table 3. Pairwise comparisons of select marburgvirus nucleoprotein sequences
Nucleoprotein (NP) pairwise comparisons of select marburgvirus sequences representing each of the major phylogenetic clades.

	**1**	**2**	**3**	**4**	**5**	**6**	**7**	**8**	**9**	**10**	**11**	**12**
**1**		**99.9**	**94.4**	**80.9**	**94.4**	**94.1**	**94.5**	**95.0**	**95.1**	**94.4**	**94.8**	**94.4**
**2**	99.4		**94.2**	**80.7**	**94.2**	**94.0**	**94.4**	**94.8**	**95.0**	**94.2**	**94.7**	**94.2**
**3**	85.6	85.5		**84.2**	**98.0**	**97.8**	**98.3**	**98.3**	**98.3**	**97.8**	**98.3**	**98.1**
**4**	85.4	85.2	93.9		**84.6**	**84.7**	**85.2**	**84.6**	**84.7**	**84.0**	**84.7**	**84.9**
**5**	85.7	85.6	94.9	97.2		**99.3**	**99.4**	**97.8**	**97.8**	**97.7**	**98.1**	**98.3**
**6**	85.6	85.5	94.7	97.1	99.4		**99.6**	**97.7**	**98.0**	**97.6**	**98.0**	**98.1**
**7**	85.7	85.6	95.0	97.5	98.9	98.9		**98.1**	**98.4**	**98.0**	**98.4**	**98.6**
**8**	85.7	85.6	95.3	93.7	94.8	94.7	94.7		**99.4**	**98.1**	**98.7**	**98.3**
**9**	85.5	85.4	95.4	93.5	94.5	94.4	94.5	98.6		**98.1**	**98.7**	**98.3**
**10**	86.2	86.0	94.1	93.5	94.3	94.2	94.5	94.2	93.8		**99.0**	**99.0**
**11**	86.3	86.0	94.2	93.8	94.5	94.3	94.7	94.3	94.0	97.9		**99.3**
**12**	85.9	85.8	93.9	93.7	94.1	94.0	94.4	94.2	93.8	98.0	97.6	

1: Rav Ken 1980, 2: 982 bat Uga 2008, 3: Musoke Kenya 1980, 4: SLAB4104Koebat SL 2017, 5: 371 bat Uga 2007, 6: 01DRC 1999, 7: Oz Zim 1975, 8: Popp Uga/Ger 1967, 9: 422Kabale Uga 2012, 10: SLAB3960Kakbat SL 2017, 11: 1379c Ang 2005, 12: 1000Kasbat SL 2018
Lightface type: % nucleotide identity; boldface type: % amino acid identity

Supplementary Table 4. Pairwise comparisons of select marburgvirus viral protein 35 sequences
Viral protein 35 (VP35) pairwise comparisons of select marburgvirus sequences representing each of the major phylogenetic clades.

	**1**	**2**	**3**	**4**	**5**	**6**	**7**	**8**	**9**	**10**	**11**	**12**
**1**		**100.0**	**94.8**	**95.1**	**94.8**	**94.8**	**95.1**	**94.5**	**95.1**	**94.5**	**94.5**	**94.2**
**2**	99.6		**94.8**	**95.1**	**94.8**	**94.8**	**95.1**	**94.5**	**95.1**	**94.5**	**94.5**	**94.2**
**3**	85.6	85.6		**99.7**	**99.4**	**99.4**	**99.7**	**98.2**	**98.8**	**98.5**	**98.5**	**98.2**
**4**	85.8	85.8	95.9		**99.7**	**99.7**	**100.0**	**98.5**	**99.1**	**98.8**	**98.8**	**98.5**
**5**	84.8	84.8	95.7	98.1		**100.0**	**99.7**	**98.2**	**98.8**	**98.5**	**98.5**	**98.2**
**6**	85.1	85.1	95.9	98.5	99.6		**99.7**	**98.2**	**98.8**	**98.5**	**98.5**	**98.2**
**7**	85.5	85.5	96.5	98.7	99.0	99.2		**98.5**	**99.1**	**98.8**	**98.8**	**98.5**
**8**	85.2	85.2	96.0	94.1	94.0	94.2	94.8		**99.4**	**97.3**	**97.3**	**97.0**
**9**	85.3	85.3	95.9	94.2	94.1	94.3	94.9	97.9		**97.9**	**97.9**	**97.6**
**10**	84.3	84.3	95.6	93.6	93.7	93.8	94.2	93.8	93.7		**98.8**	**99.7**
**11**	84.7	84.7	96.1	94.0	94.3	94.5	94.7	94.4	94.5	97.7		**98.5**
**12**	84.8	84.8	95.5	93.3	93.6	93.7	93.9	93.7	93.4	98.1	97.6	

1: Rav Ken 1980, 2: 982 bat Uga 2008, 3: Musoke Kenya 1980, 4: SLAB4104Koebat SL 2017, 5: 371 bat Uga 2007, 6: 01DRC 1999, 7: Oz Zim 1975, 8: Popp Uga/Ger 1967, 9: 422Kabale Uga 2012, 10: SLAB3960Kakbat SL 2017, 11: 1379c Ang 2005, 12: 1000Kasbat SL 2018
Lightface type: % nucleotide identity; boldface type: % amino acid identity

Supplementary Table 5. Pairwise comparisons of select marburgvirus viral protein 40 sequences
Viral protein 40 (VP40) pairwise comparisons of select marburgvirus sequences representing each of the major phylogenetic clades.

	**1**	**2**	**3**	**4**	**5**	**6**	**7**	**8**	**9**	**10**	**11**	**12**
**1**		**100.0**	**98.7**	**98.0**	**98.3**	**98.3**	**98.7**	**98.3**	**98.7**	**98.7**	**99.0**	**98.7**
**2**	99.8		**98.7**	**98.0**	**98.3**	**98.3**	**98.7**	**98.3**	**98.7**	**98.7**	**99.0**	**98.7**
**3**	86.0	86.2		**98.7**	**98.7**	**98.7**	**99.0**	**99.7**	**100.0**	**100.0**	**99.7**	**100.0**
**4**	86.5	86.7	94.3		**98.7**	**98.7**	**99.0**	**98.3**	**98.7**	**98.7**	**98.3**	**98.7**
**5**	87.3	87.5	94.7	96.9		**100.0**	**99.7**	**98.3**	**98.7**	**98.7**	**98.3**	**98.7**
**6**	87.2	87.4	94.5	96.7	99.3		**99.7**	**98.3**	**98.7**	**98.7**	**98.3**	**98.7**
**7**	87.5	87.7	95.2	97.6	99.1	98.9		**98.7**	**99.0**	**99.0**	**98.7**	**99.0**
**8**	85.9	85.9	96.1	94.4	94.6	94.4	95.1		**99.7**	**99.7**	**99.3**	**99.7**
**9**	86.5	86.5	95.6	93.9	94.3	94.1	95.0	98.1		**100.0**	**99.7**	**100.0**
**10**	85.5	85.7	95.0	93.6	94.5	94.8	94.7	95.0	94.7		**99.7**	**100.0**
**11**	85.9	86.1	94.6	93.5	94.0	94.3	94.8	94.8	94.6	97.3		**99.7**
**12**	86.5	86.7	95.4	93.8	94.4	94.5	95.1	95.1	94.8	98.1	97.1	

1: Rav Ken 1980, 2: 982 bat Uga 2008, 3: Musoke Kenya 1980, 4: SLAB4104Koebat SL 2017, 5: 371 bat Uga 2007, 6: 01DRC 1999, 7: Oz Zim 1975, 8: Popp Uga/Ger 1967, 9: 422Kabale Uga 2012, 10: SLAB3960Kakbat SL 2017, 11: 1379c Ang 2005, 12: 1000Kasbat SL 2018
Lightface type: % nucleotide identity; boldface type: % amino acid identity

Supplementary Table 6. Pairwise comparisons of select marburgvirus glycoprotein sequences
Glycoprotein (GP) pairwise comparisons of select marburgvirus sequences representing each of the major phylogenetic clades.

	**1**	**2**	**3**	**4**	**5**	**6**	**7**	**8**	**9**	**10**	**11**	**12**
**1**		**98.8**	**78.1**	**77.2**	**77.2**	**77.2**	**77.2**	**77.7**	**78.0**	**77.8**	**77.8**	**78.6**
**2**	99.3		**77.5**	**76.8**	**76.8**	**76.8**	**76.8**	**77.2**	**77.5**	**77.2**	**77.2**	**78.0**
**3**	78.1	78.0		**91.5**	**91.2**	**91.5**	**91.3**	**93.5**	**93.1**	**92.2**	**92.8**	**93.7**
**4**	78.0	77.8	91.7		**95.6**	**96.5**	**96.2**	**91.9**	**91.0**	**89.7**	**91.0**	**91.6**
**5**	78.2	77.8	91.3	95.8		**98.7**	**97.8**	**91.2**	**90.6**	**90.6**	**91.3**	**91.8**
**6**	78.2	77.9	91.2	96.0	98.8		**98.2**	**91.5**	**90.9**	**90.3**	**91.3**	**91.9**
**7**	78.4	78.0	91.6	96.3	98.1	98.2		**91.5**	**91.2**	**90.3**	**91.3**	**91.9**
**8**	78.7	78.6	93.5	92.4	91.8	91.9	92.4		**97.4**	**91.8**	**93.0**	**93.5**
**9**	79.0	78.9	93.1	91.5	91.2	91.2	91.7	97.6		**91.8**	**91.9**	**93.0**
**10**	77.9	77.7	91.4	89.7	90.0	89.7	90.0	91.1	90.8		**94.9**	**94.9**
**11**	78.2	78.0	92.4	91.0	91.0	90.7	91.0	92.4	91.6	94.9		**97.5**
**12**	78.3	78.1	92.6	91.1	91.1	91.2	91.4	92.6	91.7	95.8	97.0	

1: Rav Ken 1980, 2: 982 bat Uga 2008, 3: Musoke Kenya 1980, 4: SLAB4104Koebat SL 2017, 5: 371 bat Uga 2007, 6: 01DRC 1999, 7: Oz Zim 1975, 8: Popp Uga/Ger 1967, 9: 422Kabale Uga 2012, 10: SLAB3960Kakbat SL 2017, 11: 1379c Ang 2005, 12: 1000Kasbat SL 2018
Lightface type: % nucleotide identity; boldface type: % amino acid identity

Supplementary Table 7. Pairwise comparisons of select marburgvirus viral protein 30 sequences
Viral protein 30 (VP30) pairwise comparisons of select marburgvirus sequences representing each of the major phylogenetic clades.

	**1**	**2**	**3**	**4**	**5**	**6**	**7**	**8**	**9**	**10**	**11**	**12**
**1**		**100.0**	**90.0**	**90.0**	**92.2**	**91.8**	**91.8**	**90.4**	**90.7**	**90.7**	**91.1**	**91.1**
**2**	99.5		**90.0**	**90.0**	**92.2**	**91.8**	**91.8**	**90.4**	**90.7**	**90.7**	**91.1**	**91.1**
**3**	83.1	83.6		**94.3**	**95.7**	**95.4**	**95.7**	**95.4**	**95.7**	**97.5**	**98.2**	**98.2**
**4**	83.2	83.7	93.7		**97.5**	**97.2**	**97.9**	**94.3**	**95.0**	**95.4**	**96.1**	**96.1**
**5**	84.0	84.5	94.1	96.8		**99.6**	**99.6**	**95.4**	**95.7**	**96.8**	**97.5**	**97.5**
**6**	83.8	84.0	94.3	96.8	99.1		**99.3**	**95.0**	**95.4**	**96.4**	**97.2**	**97.2**
**7**	83.7	84.2	94.9	97.6	98.9	99.2		**95.4**	**95.7**	**96.8**	**97.5**	**97.5**
**8**	82.6	83.1	95.4	92.2	92.6	92.9	93.4		**98.6**	**96.1**	**96.8**	**96.8**
**9**	83.1	83.6	95.7	92.8	93.1	93.4	94.0	97.8		**96.8**	**97.5**	**97.5**
**10**	83.2	83.7	94.7	92.3	92.3	92.8	93.1	93.6	94.2		**99.3**	**99.3**
**11**	83.1	83.6	95.3	92.8	93.3	93.7	94.1	94.1	94.4	97.9		**100.0**
**12**	83.7	84.2	95.4	92.6	92.8	93.3	93.6	94.3	94.4	98.1	97.9	

1: Rav Ken 1980, 2: 982 bat Uga 2008, 3: Musoke Kenya 1980, 4: SLAB4104Koebat SL 2017, 5: 371 bat Uga 2007, 6: 01DRC 1999, 7: Oz Zim 1975, 8: Popp Uga/Ger 1967, 9: 422Kabale Uga 2012, 10: SLAB3960Kakbat SL 2017, 11: 1379c Ang 2005, 12: 1000Kasbat SL 2018
Lightface type: % nucleotide identity; boldface type: % amino acid identity

Supplementary Table 8. Pairwise comparisons of select marburgvirus viral protein 24 sequences
Viral protein 24 (VP24) pairwise comparisons of select marburgvirus sequences representing each of the major phylogenetic clades.

	**1**	**2**	**3**	**4**	**5**	**6**	**7**	**8**	**9**	**10**	**11**	**12**
**1**		**100.0**	**96.0**	**96.4**	**95.3**	**95.7**	**96.0**	**95.7**	**96.0**	**96.4**	**96.0**	**96.4**
**2**	99.6		**96.0**	**96.4**	**95.3**	**95.7**	**96.0**	**95.7**	**96.0**	**96.4**	**96.0**	**96.4**
**3**	86.7	86.4		**99.6**	**98.4**	**98.8**	**99.2**	**98.8**	**99.2**	**99.6**	**98.8**	**99.6**
**4**	86.9	86.5	95.4		**98.8**	**99.2**	**99.6**	**99.2**	**99.6**	**100.0**	**99.2**	**100.0**
**5**	85.7	85.3	94.5	96.6		**99.6**	**99.2**	**98.0**	**98.4**	**98.8**	**98.0**	**98.8**
**6**	86.4	86.0	95.0	97.8	98.8		**99.6**	**98.4**	**98.8**	**99.2**	**98.4**	**99.2**
**7**	86.9	86.5	95.5	98.3	98.3	99.5		**98.8**	**99.2**	**99.6**	**98.8**	**99.6**
**8**	86.6	86.5	97.0	95.7	94.6	95.5	96.1		**98.8**	**99.2**	**98.4**	**99.2**
**9**	86.7	86.6	97.1	95.5	94.5	95.4	95.9	99.3		**99.6**	**98.8**	**99.6**
**10**	86.5	86.4	95.5	94.9	94.0	94.9	95.4	96.1	96.2		**99.2**	**100.0**
**11**	86.1	86.0	94.9	94.5	93.3	94.2	94.8	95.1	95.3	98.0		**99.2**
**12**	86.6	86.7	95.4	95.0	94.1	95.0	95.5	95.9	96.1	98.8	97.6	

1: Rav Ken 1980, 2: 982 bat Uga 2008, 3: Musoke Kenya 1980, 4: SLAB4104Koebat SL 2017, 5: 371 bat Uga 2007, 6: 01DRC 1999, 7: Oz Zim 1975, 8: Popp Uga/Ger 1967, 9: 422Kabale Uga 2012, 10: SLAB3960Kakbat SL 2017, 11: 1379c Ang 2005, 12: 1000Kasbat SL 2018
Lightface type: % nucleotide identity; boldface type: % amino acid identity

Supplementary Table 9. Pairwise comparisons of marburgvirus polymerase protein sequences
Polymerase (L) protein pairwise comparisons of select marburgvirus sequences representing each of the major phylogenetic clades.

	**1**	**2**	**3**	**4**	**5**	**6**	**7**	**8**	**9**	**10**	**11**	**12**
**1**		**99.2**	**88.7**	**87.9**	**87.9**	**88.0**	**88.2**	**87.6**	**88.2**	**88.5**	**88.7**	**88.6**
**2**	99.3		**88.5**	**87.8**	**87.6**	**87.7**	**88.0**	**87.3**	**87.9**	**88.2**	**88.4**	**88.2**
**3**	83.2	83.0		**95.2**	**96.1**	**96.1**	**96.4**	**96.1**	**96.7**	**96.6**	**96.8**	**96.7**
**4**	82.5	82.4	93.4		**97.5**	**97.6**	**97.9**	**94.8**	**95.5**	**95.4**	**95.5**	**95.5**
**5**	82.8	82.6	94.2	96.5		**99.3**	**99.1**	**95.6**	**96.3**	**95.9**	**96.3**	**96.1**
**6**	82.9	82.6	94.3	96.7	99.2		**99.3**	**95.8**	**96.4**	**96.0**	**96.4**	**96.2**
**7**	82.9	82.6	94.5	97.0	98.5	98.7		**95.9**	**96.6**	**96.2**	**96.6**	**96.4**
**8**	82.5	82.3	95.1	93.0	93.7	93.8	94.0		**98.5**	**96.0**	**96.2**	**96.0**
**9**	82.5	82.3	95.1	92.9	93.6	93.7	93.9	98.1		**96.5**	**96.8**	**96.5**
**10**	82.8	82.7	94.3	92.8	93.5	93.5	93.6	93.8	93.7		**98.4**	**98.5**
**11**	82.6	82.5	94.7	93.1	93.8	93.9	93.9	94.0	94.0	97.1		**98.5**
**12**	83.0	82.9	94.7	93.0	93.7	93.7	93.8	93.8	93.8	97.9	97.2	

1: Rav Ken 1980, 2: 982 bat Uga 2008, 3: Musoke Kenya 1980, 4: SLAB4104Koebat SL 2017, 5: 371 bat Uga 2007, 6: 01DRC 1999, 7: Oz Zim 1975, 8: Popp Uga/Ger 1967, 9: 422Kabale Uga 2012, 10: SLAB3960Kakbat SL 2017, 11: 1379c Ang 2005, 12: 1000Kasbat SL 2018
Lightface type: % nucleotide identity; boldface type: % amino acid identity

Supplementary Table 10. Summary of MARV diagnostic results by sex and age class
Summary of *Rousettus aegyptiacus* real-time RT-PCR (PCR+) and virus isolation testing by sex and age class for all capture sites (n = 435) and MARV-specific antibody testing (Ab+) for Kasewe cave-Tailu village locations only (n = 140). Sera for antibody testing were not collected from ERBs captured at Kakuya and Koema cave locations. Prevalence of antibody reactive against SOSV is shown in parentheses.

		**All Sites**			**Kasewe and Tailu only**	
**Sex**	**Age Class**	**No. tested**	**PCR +**	**MARV isolations**	**No. tested**	**Ab + (%prev)**
**Female**	Adult	109	--	--	14	2
	Juvenile	132	6	3	35	3
**Male**	Adult	99	--	--	23	10
	Juvenile	95	5	1	68	9
	All adult	208	--	--	37	12 (32.4%)
	All juvenile	227	11	4	103	12 (11.7%)
**Totals**		**435**	**11**	**4**	**140**	**24**
