# Supplementary material for: Isolation of Angola-like Marburg virus from Egyptian rousette bats from West Africa
Source: Nat Commun. 2020 Jan 24;11:510. doi: 10.1038/s41467-020-14327-8 (PMC6981187; doi:10.1038/s41467-020-14327-8)
Supplement: Supplementary file 3 — Reporting Summary [file 41467_2020_14327_MOESM3_ESM.pdf]

nature research

Corresponding author(s): Tracey Goldstein, Jonathan S. Towner, Aiah Lebbie

Last updated by author(s): Dec 17, 2019

Reporting Summary

Nature Research wishes to improve the reproducibility of the work that we publish. This form provides structure for consistency and transparency in reporting. For further information on Nature Research policies, see Authors & Referees and the Editorial Policy Checklist .

Statistics

For all statistical analyses, confirm that the following items are present in the figure legend, table legend, main text, or Methods section.

n/a	Confirmed	
☐	☒	The exact sample size (*n*) for each experimental group/condition, given as a discrete number and unit of measurement
☐	☒	A statement on whether measurements were taken from distinct samples or whether the same sample was measured repeatedly
☐	☒	The statistical test(s) used AND whether they are one- or two-sided<br>*Only common tests should be described solely by name; describe more complex techniques in the Methods section.*
☒	☐	A description of all covariates tested
☒	☐	A description of any assumptions or corrections, such as tests of normality and adjustment for multiple comparisons
☒	☐	A full description of the statistical parameters including central tendency (e.g. means) or other basic estimates (e.g. regression coefficient) AND variation (e.g. standard deviation) or associated estimates of uncertainty (e.g. confidence intervals)
☒	☐	For null hypothesis testing, the test statistic (e.g. *F*, *t*, *r*) with confidence intervals, effect sizes, degrees of freedom and *P* value noted<br>*Give P values as exact values whenever suitable.*
☒	☐	For Bayesian analysis, information on the choice of priors and Markov chain Monte Carlo settings
☒	☐	For hierarchical and complex designs, identification of the appropriate level for tests and full reporting of outcomes
☒	☐	Estimates of effect sizes (e.g. Cohen's *d*, Pearson's *r*), indicating how they were calculated

Our web collection on statistics for biologists contains articles on many of the points above.

Software and code

Policy information about availability of computer code

Data collection	No software was used
Data analysis	SPSS Statistics v25 (IBM Corp, Armonk, NY), PhyML 3.0.

For manuscripts utilizing custom algorithms or software that are central to the research but not yet described in published literature, software must be made available to editors/reviewers. We strongly encourage code deposition in a community repository (e.g. GitHub). See the Nature Research guidelines for submitting code & software for further information.

Data

Policy information about availability of data

All manuscripts must include a data availability statement. This statement should provide the following information, where applicable:

- Accession codes, unique identifiers, or web links for publicly available datasets
- A list of figures that have associated raw data
- A description of any restrictions on data availability

The authors declare that all data supporting the findings of this study are available within the article and its supplementary information files, or from the authors upon request.

Field-specific reporting

Please select the one below that is the best fit for your research. If you are not sure, read the appropriate sections before making your selection.

☐ Life sciences ☐ Behavioural & social sciences ☒ Ecological, evolutionary & environmental sciences

Ecological, evolutionary & environmental sciences study design

All studies must disclose on these points even when the disclosure is negative.

Study description	A total of 435 Rousettus aegyptiacus bats were collected as a part of a joint filovirus surveillance effort in Sierra Leone. Of these, 11 tested positive for marburgvirus RNA. Virus isolates were obtained and phylogenetic analysis identified Gabon and Angola-like marburgvirus strains circulating in R. aegyptiacus bat populations in Sierra Leone.
Research sample	The research sample is 435 Rousettus aegyptiacus bats captured at four locations. These bats were collected opportunistically as part of a larger filovirus surveillance effort in Sierra Leone. The samples consisted of adult males (n=99), adult females (n=109), juvenile males (n=95), and juvenile females (n=132).
Sampling strategy	Sample size was not determined statistically. The bats were collected opportunistically as part of a larger filovirus surveillance effort in Sierra Leone. This sample size represents the the total number of Rousettus aegyptiacus collected during this surveillance effort at the time of the writing of this manuscript.
Data collection	The capture data (species identification, sex, age) were collected by field teams. These data are collected by measuring, weighing, and visually or by dichotomous keys, identifying the bat. Sexual identification is done by looking for external sexual characteristics. Bat species field identifications were confirmed on select MARV positive bats using mtDNA barcoding of the cytochrome b and cytochrome oxidase subunit 1 mitochondrial genes by laboratory teams. Diagnostic testing data, virus sequence data, and phylogenetic output were all performed by laboratory teams.
Timing and spatial scale	Rousettus aegyptiacus collection occurred as part of a larger surveillance effort beginning in January 2016 and is currently ongoing. Sampling occurs during the dry seasons and based on the availability of personnel, for a duration of 2 - 23 days. For this manuscript, sampling occurred on the following dates: Jan. 14-25 2016; 30 Jan. - 22 Feb. 2017, 23 Oct. - 12 Nov. 2017; 6 and 12 Dec. 2017; 25 Sept. - 16 Oct. 2018.
Data exclusions	No data were excluded from the analysis.
Reproducibility	For the statistical analysis, the Pearson's chi-squared tests were successfully repeated. Sequence generation from the identical sequences were successfully repeated. Phylogenetic analysese were successfully repeated. Diagnostic results were successfully repeated and confirmed through testing of additional tissues.
Randomization	This is not relevant to our study. We are describing the findings of a filovirus surveillance effort.
Blinding	Blinding is not relevant to our study. We are describing the findings of a filovirus surveillance effort.

Did the study involve field work? ☒ Yes ☐ No

Field work, collection and transport

Field conditions	Fieldwork was performed at night, typically in the absence of rainfall. Nighttime temperatures in Sierra Leone run between 22/24 °C. Sierra Leone is tropical so humidity levels are typically high. Mist netting for bats occured in forested areas, usually near cave openings.
Location	Bats were captured at four sites in the Sierra Leone districts of Moyamba (Kasewe Cave; 8°19'26.80"N 12°10'36.00"W), Kailahun (Tailu Village; 8° 18.919'N 10° 30.971'W), Koinadugu (Kakuya Cave; 9°41'24.00"N 11°40'12.00"W), and Kono (Koema Cave; 8° 52'12.00"N 10°48'36.00").
Access and import/export	All animal sampling and sample exporting was performed with permission from the Ministry of Agriculture, Forestry, and Food Security, with approval of both the Institutional Animal Care and Use Committees (IACUC) at the University of California, Davis (protocol number: 16048) and the CDC (protocol number: 2943AMMMULX). All captures were identified prior to sampling to identify any threatened and endangered species.
Disturbance	This study created only a minimal and short term disturbance at the trapping locations. This disturbance was minimized by trapping these locations only up to twice per year using trapping bouts of less than 3 weeks duration. Rousettus aegyptiacus are listed on the IUCN red list website (https://www.iucnredlist.org/search?query=rousettus%20aegyptiacus&searchType=species) as a stable species of least concern.

Reporting for specific materials, systems and methods

We require information from authors about some types of materials, experimental systems and methods used in many studies. Here, indicate whether each material, system or method listed is relevant to your study. If you are not sure if a list item applies to your research, read the appropriate section before selecting a response.

Materials & experimental systems

n/a	Involved in the study
☒	☐ Antibodies
☒	☐ Eukaryotic cell lines
☒	☐ Palaeontology
☐	☒ Animals and other organisms
☒	☐ Human research participants
☒	☐ Clinical data

Methods

n/a	Involved in the study
☒	☐ ChIP-seq
☒	☐ Flow cytometry
☒	☐ MRI-based neuroimaging

Animals and other organisms

Policy information about studies involving animals; ARRIVE guidelines recommended for reporting animal research

Laboratory animals

The study did not involve laboratory animals.

Wild animals

Animals used in this study include 435 Rousettus aegyptiacus bats captured at four locations consisting of of adult males (n=99), adult females (n=109), juvenile males (n=95), and juvenile females (n=132). These bats were collected using mist nets and were transported in breathable cotton bags and transported to a site where they were processed either by non-destructive sampling (venipuncture, oral and rectal swabs) and released at the processing site or by complete necropsy. Bats undergoing necropsy were humanely euthanized under anesthesia (isoflurane) prior to necropsy.

Field-collected samples

Diagnostic samples were inactivated in the field using MagMax lysis buffer concentrate and homogenization prior to being frozen in liquid nitrogen. Tissue and oral swab samples from bats collected at Kasewe Cave and Tailu Village were analyzed and are currently housed in country at Njala University in -80 C long-term storage. Samples collected at Kakuya and Koema Caves were processed at the University of Makeni and after inactivation were then shipped to the US (UC Davis) for diagnostics and long-term storage.

Ethics oversight

All animal sampling was performed with permission from the Ministry of Agriculture, Forestry, and Food Security, with approval of both the Institutional Animal Care and Use Committees (IACUC) at the University of California, Davis (protocol number: 16048) and the CDC (protocol number: 2943AMMMULX). All captures were identified prior to sampling to identify any threatened and endangered species.

Note that full information on the approval of the study protocol must also be provided in the manuscript.
